# A Multimodal mHealth Intervention (FeatForward) to Improve Physical Activity Behavior in Patients with High Cardiometabolic Risk Factors: Rationale and Protocol for a Randomized Controlled Trial

**DOI:** 10.2196/resprot.5489

**Published:** 2016-05-12

**Authors:** Stephen Agboola, Ramya Sita Palacholla, Amanda Centi, Joseph Kvedar, Kamal Jethwani

**Affiliations:** ^1^ Center for Connected Health Boston, MA United States; ^2^ Massachusetts General Hospital Boston, MA United States; ^3^ Harvard Medical School Boston, MA United States

**Keywords:** mobile app, physical activity, randomized clinical trial, type 2 diabetes mellitus, exercise, cardiometabolic risk factors

## Abstract

**Background:**

Physical inactivity is one of the leading risk factors contributing to the rising rates of chronic diseases and has been associated with deleterious health outcomes in patients with chronic disease conditions. We developed a mobile phone app, FeatForward, to increase the level of physical activity in patients with cardiometabolic risk (CMR) factors. This intervention is expected to result in an overall improvement in patient health outcomes.

**Objective:**

The objective of this study is to evaluate the effect of a mobile phone–based app, FeatForward, on physical activity levels and other CMR factors in patients with chronic conditions.

**Methods:**

The study will be implemented as a 2-arm randomized controlled trial with 300 adult patients with chronic conditions over a 6-month follow-up period. Participants will be assigned to either the intervention group receiving the FeatForward app and standard care versus a control group who will receive only usual care. The difference in physical activity levels between the control group and intervention group will be measured as the primary outcome. We will also evaluate the effect of this intervention on secondary measures including clinical outcome changes in global CMR factors (glycated hemoglobin, fasting blood glucose, blood pressure, waist circumference, Serum lipids, C-reactive protein), health-related quality of life, health care usage, including attendance of scheduled clinic visits and hospitalizations, usability, and satisfaction, participant engagement with the FeatForward app, physician engagement with physician portal, and willingness to engage in physical activity. Instruments that will be used in evaluating secondary outcomes include the Short-Form (SF)-12, app usability and satisfaction questionnaires, physician satisfaction questionnaire. The intention-to-treat approach will be used to evaluate outcomes. All outcomes will be measured longitudinally at baseline, midpoint (3 months), and 6 months. Our primary outcome, physical activity, will be assessed by mixed-model analysis of variance with intervention assignment as between-group factor and time as within-subject factor. A similar approach will be used to analyze continuous secondary outcomes while categorical outcomes will be analyzed by chi-square test.

**Results:**

The study is still in progress and we hope to have the results by the end of 2016.

**Conclusions:**

The mobile phone–based app, FeatForward, could lead to significant improvements in physical activity and other CMR factors in patients.

## Introduction

### Background and Significance

Physical inactivity has been identified as one of the leading risk factors contributing to the rising rates of chronic diseases [1]. Current estimates suggest that over half (52%) of adults in the United States do not meet the recommended physical activity levels [2]. Physical inactivity is an important and well-established cardiometabolic risk (CMR) factor and it has been reported that individuals with chronic diseases such as diabetes are less likely to meet current physical activity guidelines compared with the general population [3]. A number of studies to date have also confirmed the dose-response protective effect of increasing physical activity on the development of diabetes and cardiovascular disease [4,5].

The majority of the worldwide population fails to reach the recommended ≥150 minutes per week of moderate intensity exercise with “lack of time” being the most highly cited barrier to participation in sufficient physical activity in addition to a lack of motivation [5]. There is evidence that encouraging people with recently diagnosed chronic diseases such as diabetes to increase their physical activity and decrease their sedentary time may have beneficial effects on several CMR factors [6]. Therefore, it would be beneficial to develop strategies that maximize exercise adaptation, support patient goals, and offer education on health benefits of regular exercise to such patient populations.

In 2013, a dynamic text messaging intervention, TextToMove (TTM), was designed with the goals of increasing physical activity in patients with type 2 diabetes mellitus (T2DM), improving self-management of the disease, and lowering glycated hemoglobin (HbA1c) concentrations [7]. The text message (short message service, SMS) intervention program comprised of three content categories: education, feedback, and motivation. As a text messaging program, TTM was successful. However, keeping in mind, the increasing adoption of smartphones and the capability for enhanced function, we developed a more dynamic and robust mobile app, FeatForward. Additionally, there is increasing evidence that the smartphone phenomenon is reducing, as opposed to exacerbating, disparities and that smartphones are helping to bridge the digital divide among socioeconomic groups [8,9]. Therefore, FeatForward affords us the opportunity to enhance the function of the previous intervention and also extend the reach to a wider pool of patients

Our primary goal is to help users increase their level of physical activity and improve their overall health outcomes. Therefore, we hypothesize those participants using FeatForward will be more physically active and will achieve greater improvements in their CMR factors than a usual care control group that will not use the app.

### Specific Aims

Our primary aim is to evaluate the effect of FeatForward on physical activity levels. Secondary outcomes to be assessed include evaluating the effects of FeatForward on: (1) clinical outcomes by measuring changes in global CMR factors (HbA1c, fasting blood glucose, blood pressure (BP), waist circumference (WC), serum lipids, C-reactive protein (CRP)), (2) health-related quality of life, (3) health care usage, including attendance of scheduled clinic visits and hospitalizations, (4) usability and satisfaction with FeatForward, (5) participant engagement with FeatForward, and (6) physician engagement with the FeatForward physician portal, (7) continuum of behavioral regulation in exercise.

## Methods

### Trial Design

**Figure 1:**
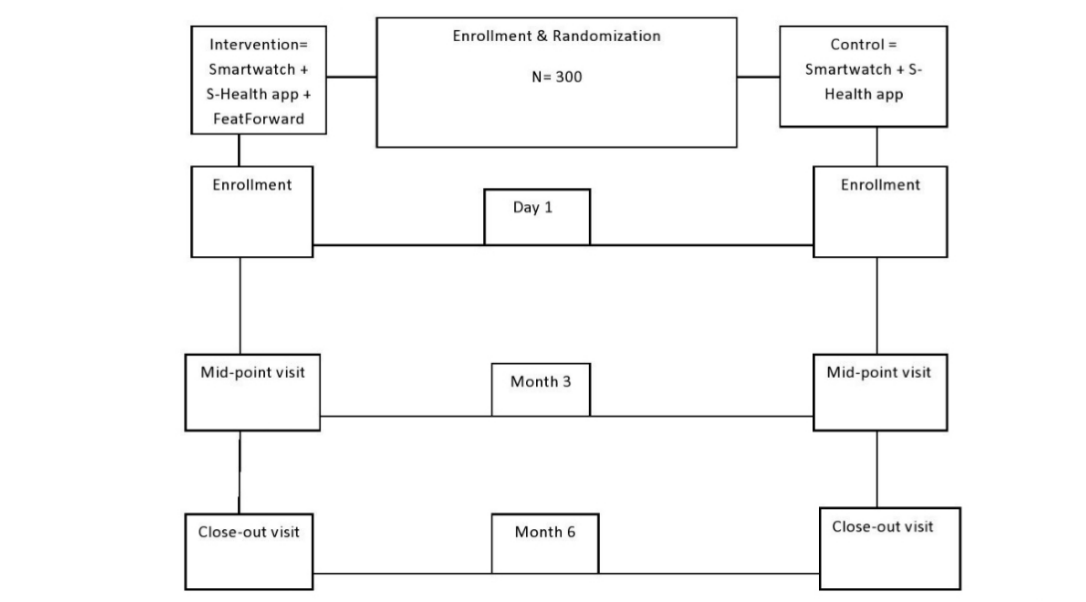
Schematic summary of the trial design.

The study will be implemented as a 2-arm randomized controlled trial (RCT) with repeated assessments at baseline, midpoint (3 months), and at the end of study (6 months). Figure 1 shows the research design.

### Participant Inclusion/ Exclusion Criteria

Patients must meet all eligibility requirements to be enrolled into the study. Eligible patients are aged 18 years or older with diagnosis of any of the following: prediabetes (HbA1c > 5.7% and body mass index (BMI) ≥ 25 kg/m^2^), T2DM (HbA1c >7.0%), prehypertension (BP of 130/90 mm Hg and family history of high BP), hypertension (BP > 140/90), and/or obesity (BMI ≥ 30 kg/m^2^). They must also be willing to attend all three study visits, able to read and speak fluent English, physically independent (ie, ability to walk without assistance), able to consent for oneself, willing to switch to a provided study smartphone and appropriate phone plan to use as their primary phone for the 6-month study duration, and willing to wear a study smartwatch during all hours excluding sleep for the duration of the study.

Ineligible patients will be defined as those who (1) have severe depression assessed by scoring ≥20 on the patient health questionnaire 8 (PHQ-8) screening questionnaire for depression, (2) have a self-reported eating disorder and/or other psychiatric disorders, (3) are currently or previously (within 3 months of enrollment) in a weight loss program, (4) had a prior or planned bariatric surgery procedure, (5) medications known to cause significant (≥5%) long-term changes in body weight or BP, (6) are pregnant or planning to get pregnant within 6 months of enrollment, (7) have a disability, dementia, or neurological deficits, and other medical or surgical conditions preventing them from engaging in self-care, (8) have serious comorbid conditions (eg, terminal cancers, end-stage renal disease) that preclude safe participation in moderate levels of physical activity, under discretion of the participants’ primary care provider.

### Recruitment Procedure

All male and female outpatients meeting the inclusion criteria will be recruited from a network of 20 primary care practices and community health centers associated with a large academic medical center in the greater Boston area. Study investigators will solicit the participation of primary care providers at these clinics and ask them to refer potentially eligible patients for the study. Potential participants undergo telephone prescreening by the study staff to ensure eligibility. This is done before any study procedure is conducted. The participants are enrolled formally only after signing the informed consent form at the enrollment visit. The participants are then asked to complete all enrollment surveys and undergo randomization procedures. All participants are typically advised to continue receiving routine medical care from physicians. Subjects in the intervention group will receive a smartphone with the FeatForward app to track physical activity and other biometric parameters, and a smartwatch. Participants in the control group will also receive a Smartphone with the sHealth app to track physical activity, and a smartwatch. All the subjects randomized to the intervention arm are taught how to use the functions of the application.

### Intervention

In collaboration with industry partner (Samsung), Partners Connected Health Innovation designed a smartphone app, FeatForward, with input from clinicians, researchers, and dieticians to help users increase their physical activity levels and lead to improvements in CMR factors. The FeatForward intervention is designed to be hyperpersonalized to respond specifically to individual users’ behavior patterns so that the app simulates an intelligent health coach partnering with users to achieve better health outcomes. The intervention also includes machine learning components for the messaging algorithm, tailoring of message frequency based on users’ activity levels, integrating patient data into the electronic medical record (EMR) through the remote monitoring data repository (our data storage infrastructure), inputs to improve the generalizability of feedback regarding health metrics (eg, weight, blood glucose), as well as a community feature to enable interactions with other similar participants and a comprehensive educational library.

#### Messaging

This features stage-specific messaging tailored to meet participants where they are in terms of their motivation level (as assessed by stage of change). While the content of the messages is tailored to a user’s stage of change, the messaging frequency and timing is uniform; all users receive two messages per day and can customize timing of these messages. There are two types of messages: motivational messages encourage physical activity, and educational messages offering information about the users’ specific medical conditions. A machine-learning algorithm will be used to select the best-fit message from a bank of messages stored in our database instead of sending random text messages that may or may not impact the participant’s behavior. These messages were developed by a team of psychologists, clinicians, and behavioral therapists. The algorithm will use the baseline data collected from participants’ at enrollment, phone usage information, and data input into app to deliver messages that are relevant to each participant. For example, if a user is meeting his/her goals on a daily basis, the program first encourages the user to set a more challenging goal. If the user then goes on to meet these new activity goals on a consistent basis, messaging responds by tailoring the content. In addition, the app algorithm is also designed to provide feedback that reflects the perceived barriers that are experienced by the user. In this way, the app mimics the role of an engaged health coach by actively monitoring and responding to the users’ progress. For example, a motivational message sent to our participants could be “A journey of a thousand miles begins with a single step. All you need to do is take that first step.”

Motivational messages are tailored to users based on their readiness to commit to behavior change. At registration, users complete a questionnaire based off of the Prochaska’s (Transtheoretical) Stage of Change theory. Users are categorized into one of the five stages of change: precontemplation, contemplation, preparation, action, or maintenance depending on their response to this questionnaire. Users then only receive motivational messages corresponding to their specific stage of change. This helps to “meet users where they are” and maximize engagement. Every 2 weeks the app’s algorithm reassesses the user’s stage of change, based off of their weekly step totals. As users move throughout the stages (both forward and backward), their messages adapt appropriately. Motivational messages also feature questions and answers to maximize engagement.

#### Coaching

The types of messages received are also tailored to the user. Educational messages only cover the medical conditions the user reports at registration (including diabetes, prediabetes, hypertension, prehypertension, and obesity). Messages are divided into modules. For example, the diabetes-related message bank includes modules on the glycemic index, blood glucose, how exercise affects blood glucose levels, diabetes-related complications, and so on. Modules include ‘quiz’ messages where users are asked to answer questions on the information they’ve received. These quiz messages help to keep the user engaged.

#### Tracking

Users are able to track their physical activity levels. They can also monitor body weight, BP, blood glucose, and heart rate using a smartwatch.

#### Community

The Community feature was designed with the aim of further educating and motivating users. Through groups, users can encourage one another and share strategies and tips that will activate or motivate health behavior change. Additionally, users in the later stages of change (preparation, action, and maintenance) can view their progress compared with users like them (based off of age, gender, etc.). This friendly competition is designed to help encourage users.

### Educational Library

All the participants need to have easy access to accurate disease-specific information to enable them to effectively self-manage their condition. Moreover, newly diagnosed patients can feel very emotional and overwhelmed as a result of being inundated with information regarding their new health condition. Many patients simply can’t process or digest new information given their emotional state, and they often don’t have the chance to review important information regarding their diagnosis with their physicians. Through the FeatForward app, users have unrestricted access to a comprehensive library with information regarding a wide array of topics that are relevant to their health condition.

#### Provider Engagement

The app syncs with a physician-facing portal that is accessible through the EMRs and includes the following additional functionalities: (1) physician access to details regarding patient activity levels and trends with optional activity reports (see Appendix 1), (2) physician ability to send self-created messages to patients through the provider portal, and (3) physician access to patient responses to messages. Not only can physicians be view patient step counts, they can also be able to send messages to individuals if they choose to do so. We believe that engaging physicians in FeatForward will also effectively engage participants and lead to improve health outcomes.

#### Depression Assessments

Because depression can impede behavior change, the app prompts users to complete the PHQ-8 on a monthly basis. Completing this survey is voluntary. If a participant chooses to complete the survey, the score will be updated in the EMR for their physician to review.

**Figure 2:**
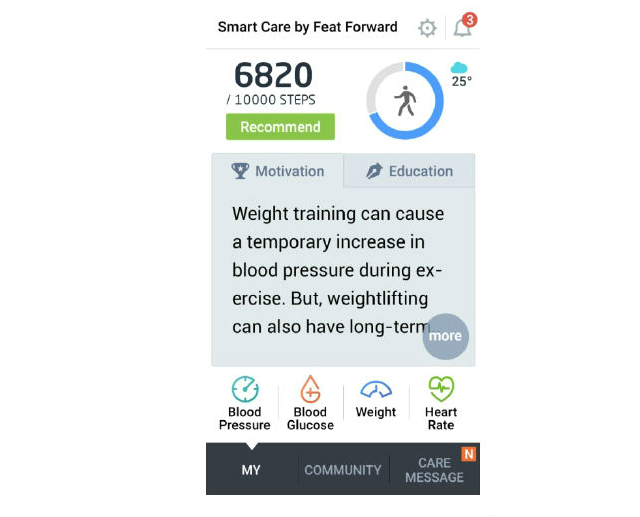
Overview of FeatForward homepage and sample message based on activity trends.

**Figure 3:**
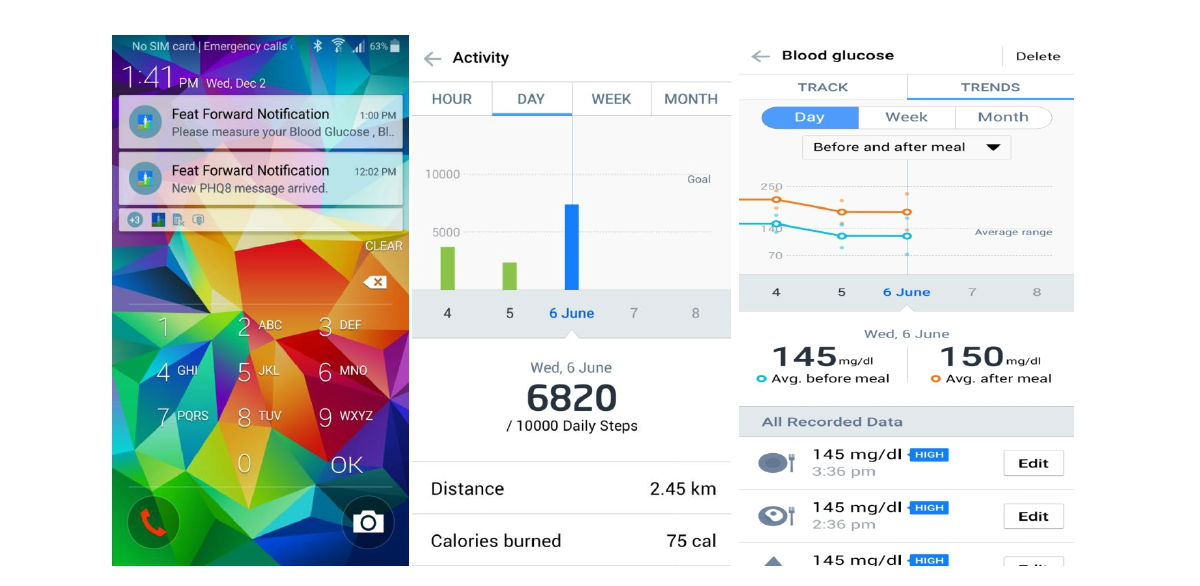
Intervention features (left to right): push notifications (reminders), physical activity tracking, and blood glucose tracking.

Screenshots of the FeatForward mobile intervention are shown in Figures 2 and 3. Figure 2 shows the homepage of the app and a sample of the educational messages. In Figure 3 , the additional features of the intervention are shown. These include push notifications, physical activity, and blood glucose tracking.

### Outcomes Measurement

The difference in physical activity levels between the control group (smartphone with sHealth app, smartwatch, and routine medical care) and intervention group (smartphone with FeatForward app, smartwatch, routine medical care) will be measured as the primary outcome of interest for this study. The participants will track physical activity using the activity trackers provided.

All secondary outcomes will be assessed at enrollment, midpoint, and at the end of the study. Body weight is measured with participants dressed in light clothing and without shoes to the nearest 0.1 kg with a validated digital scale. Changes in global CMR factors are assessed in the following ways: measurement of fasting glucose level, HbA1c, and serum lipids will occur in a standard laboratory by fasting venous blood samples collected by at baseline, the midstudy visit, and at the final study visit. Phlebotomy is performed by research nurses who will adhere to standard institutional guidelines. Additionally, high sensitivity CRP is measured at the initial visit and final study visit. BP is measured by validated automated digital BP monitors and is performed by nurses who will adhere to standard BP measurement guidelines based on American Heart Association guidelines. At each visit, the participant’s BP will be established by a minimum of two BP measurements taken at least 5 minutes apart, and the average of these readings will represent the participant’s BP. If there is greater than 5 mm Hg difference between the first and second readings, an additional reading will be obtained, and then the average of these three readings will be used to represent the participant’s BP. WC is measured at the midpoint between the iliac crest and the lowest rib. Two measurements are taken following expiration and the average of these readings will represent the participant’s WC. If there is greater than 1-cm difference between the first and second measurements, an additional measurement will be obtained, and then the average of these three measurements is used to represent the participant’s WC.

A clustered cardiometabolic risk (CCMR) score will be constructed by summing *z* scores (units of standard deviation (SD) from the population mean) of baseline values for WC, systolic blood pressure (SBP), fasting blood glucose, serum lipids, weight, HbA_1c_, using sex-specific baseline means and SDs (CCMR = (value − mean)/SD), from which *z* scores of the follow-up variables will be computed. We will divide both the mean and SD by 6, separately, to account for the number of variables included. Change in the CCMR will be calculated by subtracting the follow-up CCMR from the baseline CCMR [10]. In addition, all the CMR factors will be evaluated individually to assess the effect of the app on each of the risk factors.

The health-related quality of life will be assessed using a validated questionnaire (short form (SF)-12). Health care usage will be assessed by emergency department visits, attendance of scheduled visits and inpatient hospitalizations. These usage data will be collected from the Partners Healthcare’s Research Patient Data Registry (RPDR) at the end of the study. The RPDR is the centralized clinical data warehouse that operates under Partners Research Information Services. It securely stores all data from across Partners hospital systems in one place, and ensures security and confidentiality of patient information [11].

Usability and satisfaction will be assessed at close-out via questionnaires specifically designed for this project. Participant engagement will be measured via app usage data including overall frequency of use, frequently visited pages, time spent on these pages, responses to messages and user entries (weight, BP, glucose, heart rate). Lastly, physician engagement will be measured via time logged in the physician portal and questionnaire designed to measure portal satisfaction *.*

### Data Collection

Users can able to track their physical activity levels, body weight, BP, blood glucose, and heart rate every day. In addition, data will be collected at various time-points (baseline, months 3 and 6) as described in Table 1. All anthropometric measurements, venous blood samples, and survey data are obtained in a standardized fashion by nurses/trained research assistants. This study uses several validated and study-specific instruments for data collection in person at enrollment, midpoint (month 3) and close-out (month 6): (1) the SF-12 instrument assesses the health-related quality of life [12], (2) the usability and satisfaction questionnaires designed specifically for this study (see Appendix 2) assess usability and satisfaction, (3) the PHQ 8 screens for depression that may impede behavioral change, (4) the app usage data will be used to measure participant engagement, (5) physician portal and questionnaire designed to measure portal satisfaction will be used to measure physician engagement with FeatForward: this via time logged in the questionnaire (Appendix 3), and (6) the Behavioral Regulation in Exercise Questionnaire to measure the continuum of behavioral regulation in exercise among users [13] *.* The PHQ-8 is administered via the FeatForward app on a monthly basis and all other questionnaires along with PHQ-8 will be administered at the three study visits (see Table 1). All data collected is stored in a secure electronic database (REDCap). REDCap is secure Web application for building and managing Web-based surveys and databases. While REDCap is specifically geared to support data capture for participant enrollment and study progress it can be used to collect other types of data during the course of this study. All paper documentation including the signed consent forms will be stored in a secure cabinet, and access will be available only to institutional review board (IRB) approved study staff.

**Table 1:** Data collection schedule: the table depicts the schedule for data collection.

Intervention/control group data collection		At entry	Monthly	3 months	6 months (close-out)	Every day
**Physical activity**						X
	HbA1c	X		X	X	
	Serum lipids	X		X	X	
	Waist circumference	X		X	X	
	Blood pressure	X		X	X	
	C-reactive protein	X		X	X	
**Patient health questionnaire 8**		X	X	X	X	
**Short form-12**		X		X	X	
**Usability and satisfaction questionnaire**				X	X	
**Physician engagement questionnaire**				X	X	
**Behavioral regulation in exercise questionnaire**		X		X	X	

### Statistical Analysis Plan

#### Sample Size Estimation

A total of 300 patients (150 participants per group) will be recruited for this study. There is an 80% probability that the study will detect a treatment difference at a two-sided 0.05 significance level, if the true difference in mean step counts between the control arm and the intervention arms is 1000 steps/day. This is based on the assumption that the SD of the response variable is 2750 and accounting for an attrition rate of 20%. Participants will be followed up for a total of 6 months.

### Randomization

Following enrollment, participants will be randomized (via a computer program) to one of two groups (intervention or control) in a ratio of 1:1 (150 participants per group), using random permutated blocks to optimize balance in each treatment group at any given point in time during the study. Treatment assignment is concealed in sealed envelopes prepared by third party staff not directly involved in the study. Due to the nature of the intervention, it would be challenging to blind subjects as well as research assistants to treatment assignment but we will ensure that the data analyst(s) and study investigators will be blinded until the conclusion of the study by de-identifying all the participant data.

### Analysis

Analysis will be done using the Data Analysis and Statistical Software: STATA, version 14 with an alpha of 0.05 set a priori. We will summarize the baseline data by group assignment using descriptive statistics: means and SD will be used for continuous data with normal distribution, medians, and interquartile range for skewed data, and percentages for categorical data. Our primary outcome, physical activity, measured longitudinally over 6 months, will be assessed by mixed-model analysis of variance with intervention assignment as between-group factor and time as within-subject factor. Continuous data will be compared between control and intervention groups using the *t* -test or the Wilcoxon Mann-Whitney test and the categorical variables will be compared using the chi-square test. All analyses will be based on intention-to-treat in all randomized patients.

### Ethics and Informed Consent

Procedures of our methods have been reviewed and approved by the IRB and the study is registered at clinicaltrials.gov [NCT02551640]. The app is secure and complies with all Health Insurance Portability and Accountability Act Regulations requirements. Subjects will require a secure pass code to be able to access the app. However, if any data breach or adverse event occurs, the investigator will ensure that they are well-documented and reported according to the IRB’s requirements, regardless of causality.

For those who attend the enrollment visit, a member of the research staff will review the informed consent form with them. Details of the study, including the purpose, procedures, and expected duration will be explained to the candidate participant. Study staff will clearly communicate that the participant’s participation or nonparticipation will not affect their medical care, and that they have the right to withdraw from the study at any time. Candidate participants will be given an opportunity to ask questions about the study, and they will be informed of their right to withdraw from the study at any time. The candidate participant may then voluntarily sign and date the informed consent form, thereby providing study staff permission to enroll them in the study if they meet all inclusion criteria. If for any reason the candidate participant desires more time to consider the decision, they will be provided a copy of the unsigned consent form for reference and instructed to call the study phone number if they decide to participate in the study. All participants who sign the consent form and are screened will be documented on a screening log. All enrolled participants will be assigned a unique study identification number, and a note will be made in the source documentation verifying that the participant has willingly signed the consent form prior to participation in any study procedures in the enrollment log.

## Results

The study is still in progress and we hope to have the results by the end of 2016.

## Discussion

### Trial Implications

The study examines how to creatively apply a mHealth technology to increase and maintain physical activity in patients with chronic diseases. Our expectation is that engaging patients to take charge of their health and empowering them with essential information will lead to improved health outcomes and quality of life. The FeatForward mobile app mimics a health coach to help engage users and increase their physical activity through education, tracking, feedback, and social network. The mobile app also features a physician-facing portal, which will serve as a channel of communication between the physicians and patients to improve the overall health outcomes.

Sedentary behavior and physical inactivity have been associated with increased risk for chronic diseases [1]. In addition to being the most common CMR, physical inactivity also happens to be the easiest to target [1]. A study by Lamb and colleagues [3] found that increasing the amount of time spent being physically active and decreasing the time spent sedentary may be an important strategy for self-management of chronic diseases such as diabetes early in the course of the disease. In recent times, several patient-centered interventions have been used to engage users and help them increase their level of physical activity, thereby improving the health outcomes over time. Such interventions use mobile apps and employ a range of features including providing feedback based on physical activity tracking, providing motivational messages, demonstrating the right way to exercise, setting and monitoring personalized goals, incorporating social media, and helping users schedule their exercise regimes [7].

A trial evaluating a mobile phone app, MyBehavior, designed to track physical activity and eating behavior data with personalized feedback demonstrated that the app was associated with increased physical activity [10]. Furthermore, a recent study by Block [14] used a fully automated, algorithm-driven behavioral intervention, Alive-PD, delivered via the Web, mobile phone, and automated phone calls for diabetes prevention. The intervention was associated with improved glycemic control, and decreased body weight, BMI, WC, triglycerides/high-density lippoproteins ratio, and diabetes risk [14].

While data demonstrating the positive impact of mobile health on clinical outcomes is growing, there is however mixed evidence of the impact of digital health technologies on health care costs. Results from a recent study by Bloss et al [15] concluded that there are no differences in health care costs or usage outcomes in patients using mHealth technologies for management of chronic diseases compared with a control group that received a standard disease management program [15]. Another study, a systematic literature review *of the cost-utility and cost-effectiveness of telemedicine and mHealth systems,*  reported mixed effect that s *ome studies demonstrated that telemedicine can reduce the costs while some others showed no impact on the health care cost reduction [16].* On the other hand, a meta-analysis of 11 randomized controlled trials including 5702 patients with heart failure showed that remote monitoring facilitated by electronic devices was associated with significant decreases in health care usage and costs [17]. With capabilities such as real-time remote monitoring of patients and data sharing with care providers, digital health technologies allow early intervention for high-risk patients in need of attention and thereby prevent unforeseen health care expenses [17]. Literature on cost effectivess of mHealth technologies is sparse and further research is warranted to demonstrate the impact of mHealth applications on cost and usage outcomes in the management ofchronic diseases.

The FeatForward app features a care provider portal integrated with the EMRs that allows physicians to monitor the patients’ progress and intervene when appropriate. We believe that early intervention by care providers will lead to a decrease in the number of emergency department and unscheduled clinic visits, thereby decreasing health care costs in the long run. Furthermore, mHealth technologies foster patient engagement for self-care and enhance their knowledge about their conditions to help them make informed health care decisions. Thus, these informed patients have better outcomes than uninformed patients [16,18]. The FeatForward app provides participants with an unrestricted access to a comprehensive library with information regarding a wide range of topics that are relevant to their health. We believe that increasing participants’ awareness of their conditions will help them better manage their illnesses and lead to improved outcomes.

### Limitations

An important limitation is that the FeatForward app is designed to support only android devices for this trial. This is important because a sizeable proportion of the US population own Apple operating system (iOS) devices and this can potentially affect the study’s accrual rates. While there is no robust data about the differences in demographics or the dynamics of user preferences of iOS versus Android users, early reports suggest that loyalty rates are usually high among users of either platform. Therefore, we speculate that some eligible iOS users may be reluctant to switch to Android for the duration of the study. Additionally, the impact of this limitation on the study’s generalizability is yet to be determined but we do not envisage any significant differences in the demographics of people using either of the platforms. Despite these limitations, this study demonstrates the potential of using personalized messages and activity tracking to deliver a theory-driven intervention to users. As we move into a time where increasingly more people are employing technology to monitor their health, we believe that FeatForward holds great promise in increasing disease knowledge and improving health outcomes.

### Conclusions

Given the high prevalence of physical inactivity and chronic diseases in today’s society, findings from this study, may potentially help participants engage in healthier lifestyles and lead to decreased health care costs and improved patient health outcomes. Additionally, we hope that the research from our trial will generate data for large, multicenter trials on similar evidence-based approaches.

## Appendix

Multimedia Appendix 1 Physican Portal Sample Views.

Multimedia Appendix 2 Usability Questionnaire.

Multimedia Appendix 3 Physician Engagement Questionnaire.

## Abbreviations

BMI: body mass index
BP: blood pressure
CCMR: clustered cardiometabolic risk score
CMR: cardiometabolic risk factor
CRP: C-reactive protein
EMR: electronic medical record
HbA1c: glycated hemoglobin
iOS: Apple operating system
IRB: institutional review board
RCT: randomized controlled trial
RPDR: research patient data registry
LMR: longitudinal medical record
PHQ-8: patient health questionnaire 8
SBP: systolic blood pressure
SD: standard deviation
SF-12: short form 12
SMS: short message service
T2DM: type 2 diabetes mellitus
TTM: TextToMove
WC: waist circumference
