# Supplementary material for: A Multimodal mHealth Intervention (FeatForward) to Improve Physical Activity Behavior in Patients with High Cardiometabolic Risk Factors: Rationale and Protocol for a Randomized Controlled Trial
Source: JMIR Res Protoc. 2016 May 12;5(2):e84. doi: 10.2196/resprot.5489 (PMC4882414; doi:10.2196/resprot.5489)

Appendix 1 Physician Portal Sample Views

1.1 Physician Portal view showing monthly, weekly and daily weight and blood pressure readings

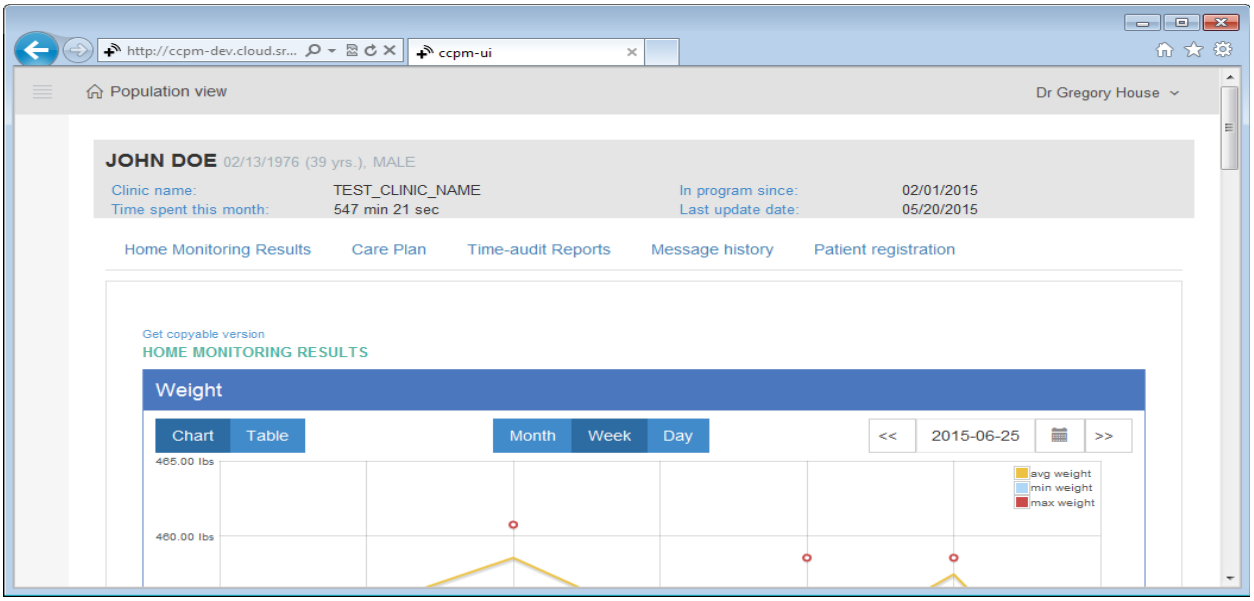

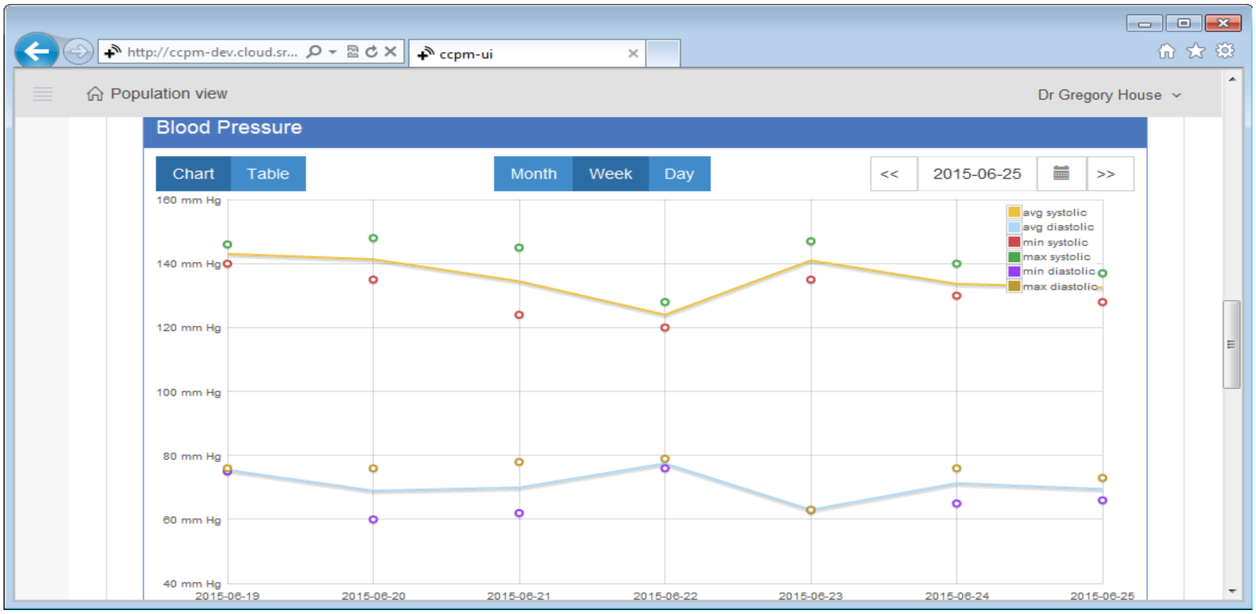

1.2 “Population View” on the Physician Portal showing a summary of data for each patient

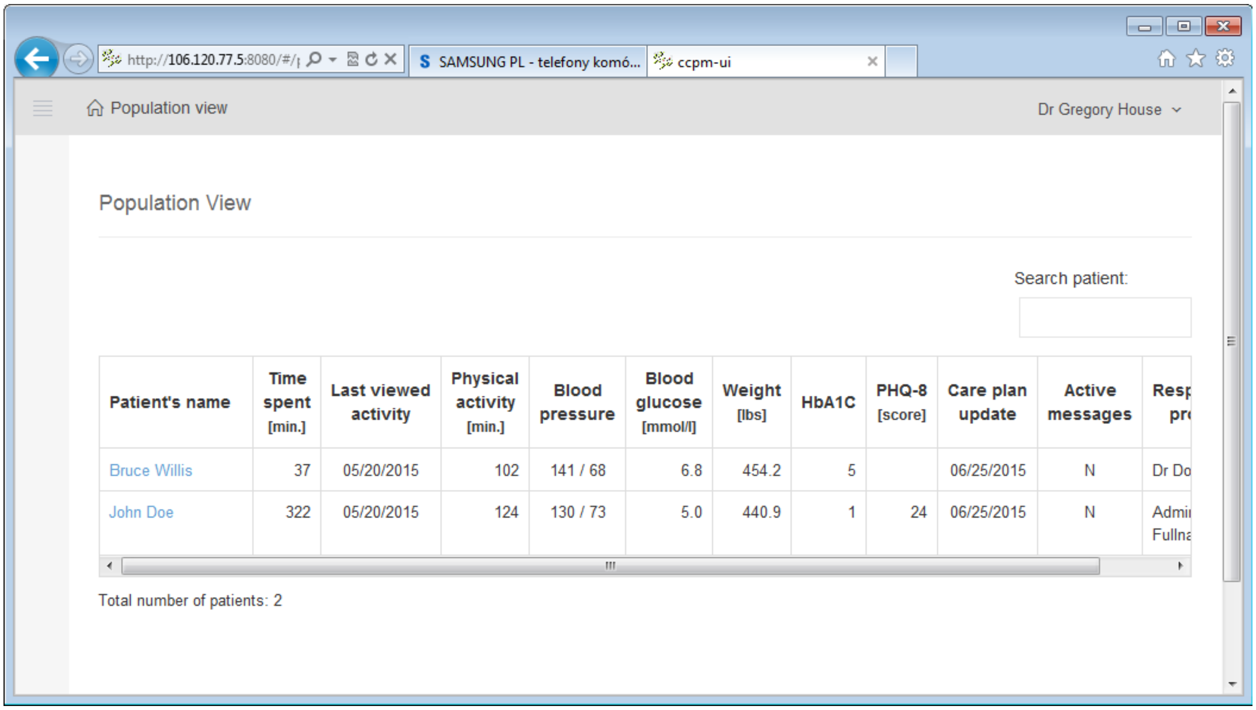

Supplement: Multimedia Appendix 1 [file resprot_v5i2e84_app1.pdf]
