# Supplementary material for: A Multimodal mHealth Intervention (FeatForward) to Improve Physical Activity Behavior in Patients with High Cardiometabolic Risk Factors: Rationale and Protocol for a Randomized Controlled Trial
Source: JMIR Res Protoc. 2016 May 12;5(2):e84. doi: 10.2196/resprot.5489 (PMC4882414; doi:10.2196/resprot.5489)
Supplement: Multimedia Appendix 2 [file resprot_v5i2e84_app2.pdf]

Appendix2: Usability Questionnaire

1. Did you use the Feat Forward app?

   A) A couple times a day
   B) Daily
   C) 2-4 times/week
   D) Weekly
   E) Biweekly
   F) Monthly
   G) Other__________
   H) Did not use the Feat Forward app

2. How useful did you find information the Feat Forward app gave you regarding your physical activity?

   A) Very useful
   B) Somewhat useful
   C) A little useful
   D) Not very useful
   E) Not useful at all

3. How useful did you find information the Gear Fit watch gave you regarding your physical activity?

   A) Very useful
   B) Somewhat useful
   C) A little useful
   D) Not very useful
   E) Not useful at all

4. Did you find using the Feat Forward app encouraged you to be more physically active?

   A) Yes a lot
   B) Yes a little
   C) No

5. Did you find using the Gear Fit watch encouraged you to be more physically active?

   A) Yes a lot
   B) Yes a little
   C) No

6. Did you discuss your participation in this study with others?

   A) Yes: With family/friends

B) Yes: With your doctor/care provider (s)
C) Yes: With both family/friend (s) and your doctor/care provider (s)
D) No- (skip next question)

7. If yes, how useful was the FeatForward app in helping start discussions about your condition and/or physical activity?

F) Very useful
G) Somewhat useful
H) A little useful
I) Not very useful
J) Not useful at all

8. Did you use the Feat Forward app to view your steps?

A) Yes
