# Supplementary material for: A Multimodal mHealth Intervention (FeatForward) to Improve Physical Activity Behavior in Patients with High Cardiometabolic Risk Factors: Rationale and Protocol for a Randomized Controlled Trial
Source: JMIR Res Protoc. 2016 May 12;5(2):e84. doi: 10.2196/resprot.5489 (PMC4882414; doi:10.2196/resprot.5489)
Supplement: Multimedia Appendix 3 [file resprot_v5i2e84_app3.pdf]

Appendix 3

Physician Engagement Questionnaire

. Did you use the Feat Forward physician portal?

A) A couple times a day
B) Daily
C) 2-4 times/week
D) Weekly
E) Biweekly
F) Monthly
G) Other__________
H) Did not use the Feat Forward physician portal

2. How useful did you find information the Feat Forward physician portal gave you regarding your patient's activity?

A) Very useful
B) Somewhat useful
C) A little useful
D) Not very useful
E) Not useful at all

3. How useful did you find information the Feat Forward physician portal gave you regarding your patient's home monitoring (e.g. blood glucose, blood pressure, weight, etc.)?

A) Very useful
B) Somewhat useful
C) A little useful
D) Not very useful
E) Not useful at all

4. How useful did you find the ability to directly message your patient(s)?

A) Very useful
B) Somewhat useful
C) A little useful
D) Not very useful
E) Not useful at all

5. Did you find using the Feat Forward physician portal kept you engaged with your patient's home care?

A) Yes a lot
B) Yes a little
C) No

6. Did you discuss the FeatForward monitoring data with your patient at their medical visits?

A) **Yes**
B) **No (skip next question)**
C) **I did not see my patient during the study time to discuss (skip next question)**

7. If yes, how useful was the FeatForward program in helping start discussions with your patient about their home monitoring and physical activity?

F) **Very useful**
G) **Somewhat useful**
H) **A little useful**
I) **Not very useful**
J) **Not useful at all**
